# Granuloma Annulare Mimicking Squamous Cell Carcinoma

**DOI:** 10.7759/cureus.27372

**Published:** 2022-07-27

**Authors:** Anna E Skrade, Chase A Pitchford, Brett C Neill, Cary Chisholm, Stanislav N Tolkachjov

**Affiliations:** 1 Dermatology, University of Missouri School of Medicine, Columbia, USA; 2 Dermatology, Epiphany Dermatology, Rockwall, USA; 3 Dermatopathology, Epiphany Dermatology, Waco, USA; 4 Mohs and Complex Facial Reconstructive Surgery, Epiphany Dermatology, Rockwall, USA

**Keywords:** mimicker, granulomatous dermatitis, advance practice professional, and misdiagnosis, -dermatopathology, cutaneous oncology, squamous cell carcinoma (scc), granuloma annulare

## Abstract

Misdiagnosing granuloma annulare (GA) for a malignant process can lead to unnecessary and costly treatment avenues for the patient. Thus, it is salient for surgeons to independently evaluate a patient’s clinical and histopathologic presentation before proceeding with surgery. We present a case of a 67-year-old male with a biopsy-proven squamous cell carcinoma (SCC) on the dorsal hand who presented for Mohs micrographic surgery (MMS). At this time, the surgeon noticed the histopathologic diagnosis did not match the patient’s clinical appearance. GA was diagnosed following a repeat biopsy of the lesion, which prevented an unnecessary Mohs procedure. We present this case primarily to highlight the importance of clinicopathologic correlation by the surgeon when a patient is referred for surgery.

## Introduction

Many clinical and histopathological mimickers of squamous cell carcinoma (SCC) have been described in the literature including hypertrophic lichen planus, tophaceous gout, and keratoacanthomas [1-3]. While SCC often requires invasive and costly interventions to optimize patient outcomes, many SCC mimickers are benign and require minimal treatment, highlighting the importance of correct diagnoses prior to unnecessary interventions. Our case provides an additional example of a benign clinicopathological mimicker of SCC in a patient with granuloma annulare (GA). 

GA is a benign cutaneous reaction pattern presenting with papules that group in an annular shape and affect patients of all ages and gender. The etiology of GA is unknown, though many potential disease associations and triggers have been described in the literature, including preceding infectious diseases and traumatic events. GA is often asymptomatic, self-limited, and requires little to no treatment when it is localized. However, when misdiagnosed for a malignant process such as SCC, patients may be referred for surgery. 

Dermatologic and Mohs micrographic surgeons often encounter lesions where the clinical presentation does not match the histopathological description or vice versa. Given there are histopathological and clinical mimickers in Mohs micrographic surgery (MMS) [1-3], surgeons must have a low threshold for re-biopsy when the clinicopathologic correlation (CPC) seems inconsistent. A clinical suspicion for further evaluation prior to surgery may result in avoidance of inappropriate surgical therapy for non-operative conditions.

We report a case of GA of the dorsal hand mimicking SCC, highlighting the importance of independent CPC evaluation of lesions referred for surgical treatment.

## Case presentation

**Figure 1:**
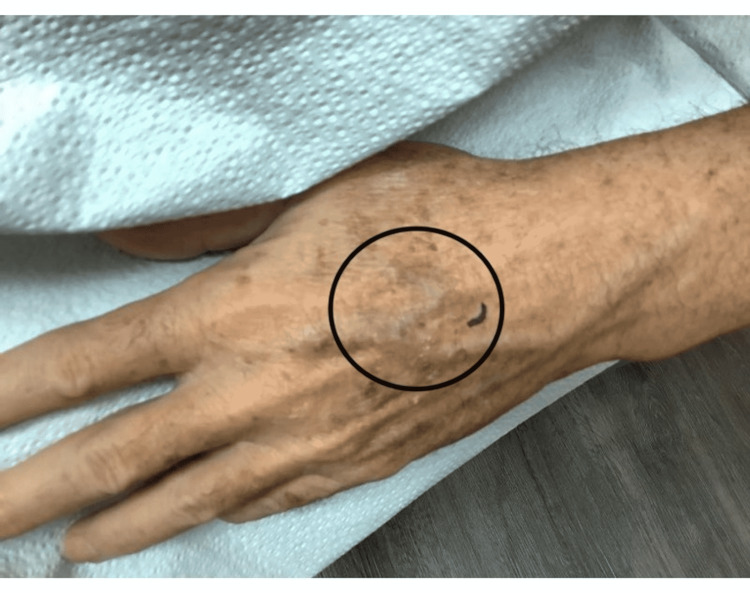
Left dorsal hand. Erythematous plaque with a raised annular border.

**Figure 2:**
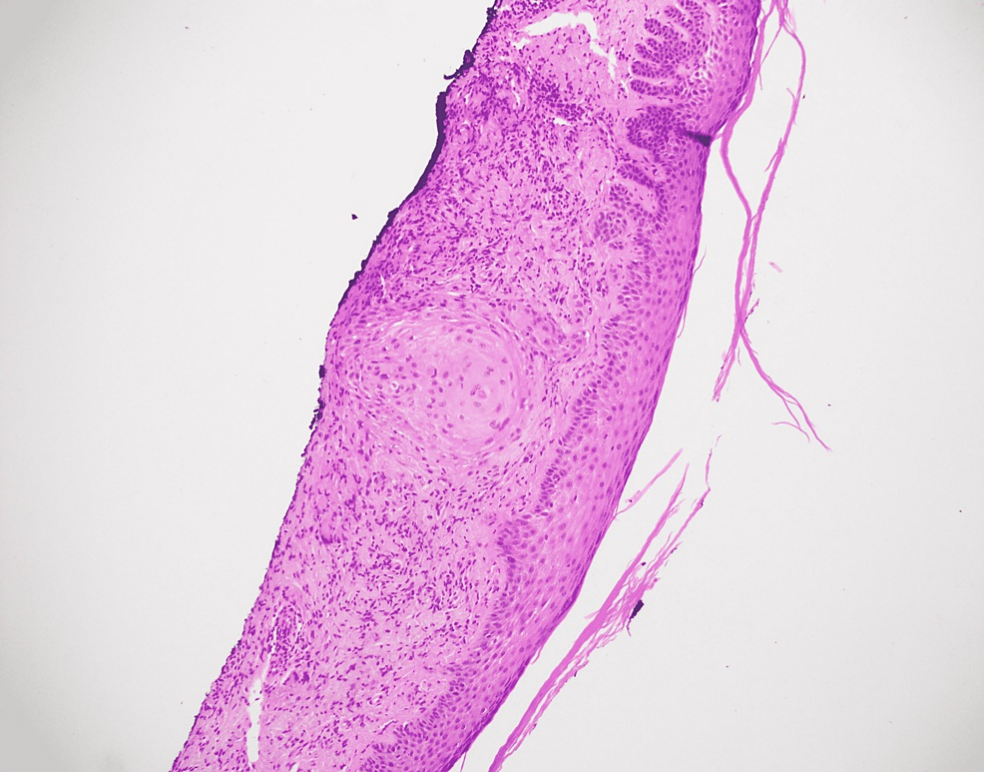
Histopathology of initial biopsy interpreted as well-differentiated squamous cell carcinoma. A small focus of invaginating well-differentiated squamous epithelium with small nests and a mild degree of atypia. (Hematoxylin-eosin stain; original magnification: 40X magnification)

**Figure 3:**
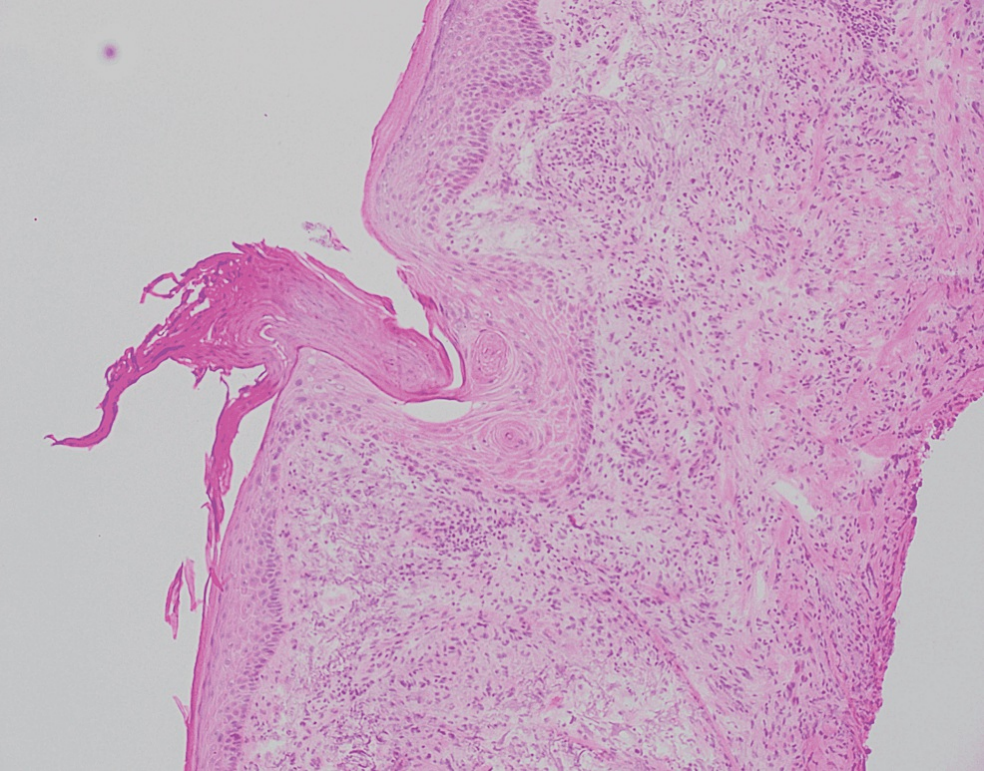
Frozen section from Mohs micrographic surgery of granuloma annulare mimicking squamous atypia. Pathology shows a keratotic center and squamous atypia. (Hematoxylin-eosin stain; original magnification: 100X magnification)

**Figure 4:**
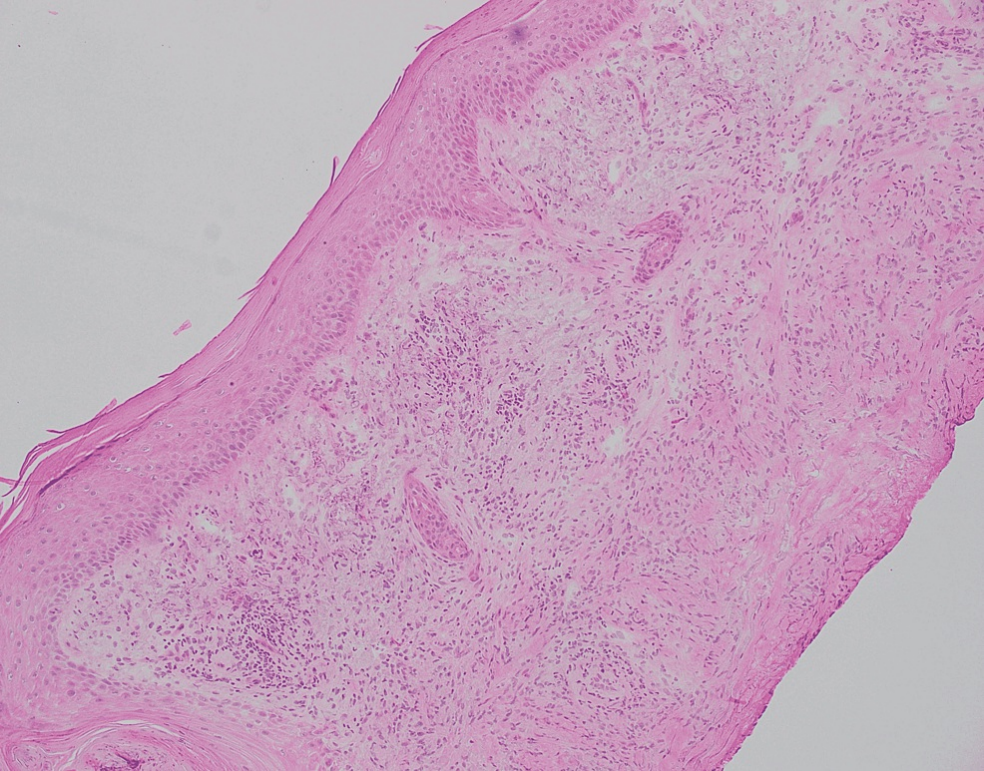
Frozen section from Mohs micrographic surgery of granuloma annulare showing mucin deposition in the dermis with a granulomatous infiltrate. (Hematoxylin-eosin stain; original magnification: 100X magnification)

A 67-year-old male with a history of basal cell carcinoma was referred for MMS for a biopsy-proven SCC on the left dorsal hand (Figure 1). The lesion had been present for two years and biopsied with a clinical suspicion for SCC. The skin biopsy showed a small focus of invaginating well-differentiated squamous epithelium with small nests and a mild degree of atypia (Figure 2). These areas had surrounding fibrous stroma with mixed inflammation. These findings were interpreted as a well-differentiated SCC by the dermatopathologist. On the day of MMS, the surgeon noticed that the histopathologic diagnosis did not match the clinical appearance which was an annular granulomatous plaque of the dorsal hand, a common presentation of GA. A repeat biopsy was obtained for frozen and permanent sections. One section of the frozen section biopsy showed some squamous atypia (Figure 3). The frozen section biopsy was consistent with GA, showing increased mucin deposition in the dermis with palisading histiocytes around a focus of necrobiosis and a perivascular lymphocytic infiltrate (Figure 4). Therefore, MMS was avoided. The permanent section pathology further supported GA.

## Discussion

Some clinicopathological mimickers of SCC that have been described are hypertrophic lichen planus [1], gouty tophi [2], and pemphigus foliaceus [4]. In addition to clinically resembling SCC, these and other inflammatory mimickers can exhibit pseudoepitheliomatous hyperplasia, often called pseudocarcinomatous hyperplasia on histology which can be difficult to distinguish from SCC. These examples highlight the importance of independent evaluation of each patient by Mohs surgeons. Their clinicopathologic training becomes valuable when evaluating a true malignancy versus a mimicker referred for surgery.

In the present case, the original biopsy showed small nests of mildly atypical squamous epithelium that appeared to infiltrate into the dermis with surrounding fibrosis and inflammation, leading to an original diagnosis of well-differentiated SCC. In retrospect, while a small well-differentiated SCC could still be possible, it is more likely to represent a focus of a benign process such as a ruptured hair follicle or focus of trauma with reactive epithelial atypia. The diagnostic features of GA were more prominent on the second biopsy, indicating that, while this could represent a collision lesion, a more common explanation involves the importance of sampling error in larger lesions.

The differential for cutaneous annular lesions in the elderly includes tinea corporis, sarcoidosis, keratoacanthoma centrifugum marginatum (KCM), actinic granuloma, and GA. Many believe KCM to be a low-grade SCC that can recur but not metastasize [5]. Features of KCM are notable hyperkeratotic borders and the development of central atrophic scars. GA is a benign cutaneous disease that classically presents as arciform to annular, nonscaly, plaques on the dorsal hands or feet, primarily in young people. The lesions are distributed in various patterns from localized, photodistributed, and occasionally generalized/disseminated. Similar to our patient, GA primarily occurs on the lateral and dorsal surfaces of the hands and feet but can occur anywhere. There are multiple forms of GA varying in clinical morphology, namely papular, perforating, macular, and deep dermal, and these and other subtypes are on a spectrum of reactive granulomatous dermatitis [6].

GA has a classic histopathologic pattern of focal degeneration of collagen and elastic fibers, mucin deposition, and a lymphohistiocytic infiltrate in the upper and mid dermis. The clinical differential is broad and is dependent on its subtype. For example, for conventional GA, annular entities including actinic granuloma and annular sarcoidosis should be considered, whereas papular GA can simulate flat warts, and perforating GA can resemble perforating calcinosis cutis or molluscum contagiosum.

As GA and reactive granulomatous dermatitis can be secondary to a systemic condition like diabetes or even cancers, diffuse or extensive involvement may lead to additional workup. Treatment of GA includes topical or intralesional corticosteroids as well as immunomodulators. Spontaneous resolution of GA occurs within two years in 50% of cases with a 40% recurrence rate [6].

As MMS is a treatment for skin cancers, referrals sent for Mohs surgery are typically malignant. However, Mohs surgeons should be aware of clinical and histopathologic mimickers of benign lesions and independently evaluate each patient with a CPC to avoid unnecessary surgery and ensure timely and appropriate treatment for patients.

## Conclusions

Many cutaneous diseases, including but not limited to GA, mimic SCC and thus warrant referral to MMS. As with the patient in this report, many of these mimickers are benign and self-limiting processes that would otherwise resolve with supportive or no treatment. Similarities in both clinical and histopathologic presentation make distinguishing benign cutaneous diseases from SCC challenging. As Mohs surgeons are uniquely trained in the clinical and pathologic presentation of cutaneous disease, it is critical each patient is evaluated with respective disease correlation upon arrival for surgery. Recognizing SCC mimickers can save patients invaluable time, decrease financial burden, and mitigate the risk accompanying surgical procedures. 
